# Navigating uncertainty in patient care: a closer look at emergency medicine residents in Brazil

**DOI:** 10.3389/fmed.2025.1578575

**Published:** 2025-09-10

**Authors:** Gabriela Boemeke, Larissa A. O. Barbosa, Rodrigo C. Menezes, Luiz F. Quintanilha, Katia M. Avena, Bruno B. Andrade

**Affiliations:** ^1^Faculdade de Minas, Belo Horizonte, Brazil; ^2^Faculdade Zarns, Salvador, Brazil; ^3^Instituto Monster de Ensino, Assistência, Pesquisa e Desenvolvimento Tecnológico em Saúde, Salvador, Brazil; ^4^Laboratório de Pesquisa Clínica e Translacional, Instituto Gonçalo Moniz, Fundação Oswaldo Cruz, Salvador, Brazil; ^5^Universidade Salvador, Salvador, Brazil; ^6^Clariens Educação, São Paulo, Brazil

**Keywords:** medical students, psychological stress, medical education, graduate medical education, emergency medicine

## Abstract

**Background:**

In emergency care, inexperience and case complexity can generate uncertainty and stress among physicians, impairing decision-making and impacting patients and the healthcare system. Despite its relevance, little is known about uncertainty among Emergency Medicine residents (EMRs).

**Aim:**

To investigate decision-making uncertainty among EMRs, identifying the most affected dimensions and the influence of sociodemographic and academic profiles.

**Methods:**

This cross-sectional study included EMRs in Brazil, regardless of residency year. Data were collected via snowball sampling using an anonymous electronic form distributed through virtual groups and email. Information on sociodemographic/academic characteristics, as well as the Physicians’ Reactions to Uncertainty (PRU) questionnaire, was obtained.

**Results:**

A total of 124 EMRs participated (median age 28 years; 52% female). The majority of the participants were first-year residents (49%), with no prior residency experience (92%) or other healthcare degrees (94%). The Southeast region was most represented (38%). The median PRU score was 49.0 (IQR: 41.0–57.0), with the highest scores observed in anxiety due to uncertainty (21.0) and reluctance to disclose uncertainty to patients (13.0). Cluster analysis identified three groups: high (43%), moderate (35%), and low (22%) uncertainty. Male and third-year residents had significantly lower total PRU scores, especially in anxiety (*p* = 0.023 and *p* = 0.017). Previous healthcare training or residency did not significantly affect uncertainty levels.

**Conclusion:**

EMRs show substantial uncertainty in decision-making, particularly in anxiety and reluctance to disclose uncertainty to patients. First-year residents and those without prior healthcare training or residency are more affected. Male and more experienced residents report lower uncertainty, especially regarding anxiety. These findings suggest that targeted interventions—such as enhanced training and structured support—could help EMRs manage uncertainty, improving both decision-making and well-being in emergency settings.

## 1 Introduction

Emergency Medicine is a relatively new specialty in Brazil, officially recognized in 2016 by the Brazilian Council of Medicine, the Brazilian Medical Residency Council, and the Brazilian Association of Medical Education (1, 2). Serving as a vital entry point for newly graduated doctors, this specialty is essential for equipping future healthcare professionals with the necessary skills to handle emergencies competently (3, 4). However, this need for competency must be understood within the broader context of medical education in Brazil, which faces significant challenges, such as the expansion of medical schools and the uneven distribution of healthcare professionals across the country (5). Despite the Brazilian Curriculum Guidelines mandating that medical courses prepare doctors for independent emergency care, there are still noticeable gaps in teaching practices within this domain, leading to suboptimal performance among new professionals (5–7).

Uncertainty is an intrinsic aspect of medical practice, affecting everyone from medical students to experienced practitioners. During residency, this challenge becomes particularly pronounced, as healthcare professionals face complex and urgent situations in emergency units that demand rapid and, at times, invasive interventions. The combination of limited experience and high clinical complexity can generate significant stress, frustration, and insecurity, ultimately affecting professional judgment and decision-making (4). These effects can, in turn, compromise patient outcomes and strain the healthcare system (8–11).

Beyond its direct impact on physicians, uncertainty can have broader systemic consequences, including excessive diagnostic testing in pursuit of certainty, heightened patient anxiety from incidental findings, unnecessary procedures, and increased healthcare costs (12, 13). Such implications underscore the need for residency programs to prepare physicians to navigate uncertainty effectively, ensuring patient safety while promoting the efficient use of healthcare resources.

While numerous studies have examined decision-making uncertainty among medical students and residents (8–10, 14, 15), research focusing on Emergency Medicine Residents (EMRs) remains limited, despite the intricate decision-making context these professionals encounter. Understanding and addressing decision-making uncertainties in this context is crucial for enhancing individual performance through the training programs and improving the overall quality of emergency care. Therefore, the present study aims to fill this knowledge gap in the field by exploring decision-making uncertainty among EMRs, identifying the most affected dimensions, and evaluating how sociodemographic and academic backgrounds can influence this phenomenon.

## 2 Methods

### 2.1 Study design and population

This study was designed as a cross-sectional analysis conducted from August 2021 to November 2021 in Brazil. The population comprised of EMRs actively enrolled in accredited residency programs nationwide. Residents of any year of training were eligible, while those with incomplete evaluation instruments or duplicate responses were excluded.

### 2.2 Sample selection

For the sample size calculation, we considered the 52 certified Emergency Medicine (EM) professionals reported in the 2020 Brazilian Medical Demography study (16), which was the most recent data available at the time of study design. Given that the Emergency Medicine residency lasts 3 years, the estimated total population was 156 residents. A minimum of 112 residents was required, considering population heterogeneity, a 5% margin of error, a 95% confidence interval (CI), and an 80% statistical power.

### 2.3 Data collection

Data were collected through an anonymous, structured electronic form that included sociodemographic and academic questions, as well as the Physicians’ Reactions to Uncertainty (PRU) questionnaire (17). The sociodemographic section gathered information on gender, age, and region of Brazil, while the academic section covered residency year [categorized as first (R1), second (R2), or third (R3)], previous health-related degrees, and any prior specializations or residencies. The PRU questionnaire (17), validated for the Brazilian population (18), comprises 15 items grouped into four dimensions: anxiety from uncertainty (5 items); concern about bad outcomes (3 items); reluctance to disclose uncertainty to patients (5 items); and reluctance to disclose mistakes to other physicians (2 items) (18). Each item is rated on a six-point Likert scale (1 = strongly disagree to 6 = strongly agree), with higher total scores indicating greater reactivity to uncertainty. As recommended by the authors, items with a positive connotation toward uncertainty were reverse-scored before calculating the domain score to ensure directional consistency. The PRU is the most commonly used scale in the literature to quantify physicians’ uncertainty in decision-making (9, 14) and has been widely applied to assess aspects related to this uncertainty in clinical decision contexts (19).

Participants were invited using a non-probabilistic snowball sampling technique, wherein initial participants recruited peers from various Emergency Medicine residency programs across Brazil (20, 21). The form was distributed via virtual groups of EMRs nationwide, identified through social media and messaging apps suggested by the residents themselves. Additionally, contacts were made with coordinators of Emergency Medicine residency programs in Brazil to disseminate the form via email, targeting residents not present in virtual groups.

### 2.4 Data analysis

The statistical analysis was performed using R Studio software, utilizing the compareGroups package to construct the tables. Not all available variables were included in the study. The following R packages were employed for statistical analysis: compareGroups (version 4.5.1), sjPlot (version 2.8.17), geobr (version 1.9.1), ggridges (version 0.5.6), cluster (version 2.1.8), pheatmap (version 1.0.12), and factoextra (version 1.0.7).

### 2.5 Statistical methods

Descriptive statistics were calculated to summarize the demographic and clinical characteristics of the participants. Continuous variables were presented as means and standard deviations, while categorical variables were presented as counts and percentages. The Mann–Whitney U test and Kruskal–Wallis test were used to compare continuous variables between groups. Median values and interquartile ranges (IQR) were reported as measures of central tendency and dispersion. Pearson’s chi-squared test was employed to compare categorical variables. These variables were displayed as counts and frequencies (%) in the tables.

Hierarchical clustering was performed to identify subgroups of residents with similar profiles in terms of their responses to uncertainty. Each domain was standardized using z-scores across all participants. Clustering was conducted using Euclidean distance and Ward. D2 linkage, and the optimal number of clusters was determined via the Silhouette method. Resulting clusters were compared across sociodemographic strata and domain scores.

To investigate the independent associations between sociodemographic and academic factors with the PRU scores, linear regression models were fitted, and their regression coefficients (*β*) with 95% confidence intervals and *p*-values were reported for each association.

### 2.6 Ethical considerations

This study followed the Declaration of Helsinki and Brazilian Health Council Resolutions 466/12 and 510/16. Furthermore, it was approved by the Brazilian Research Ethics Committee (protocol number 47109121.2.0000.5032). Data confidentiality and participant privacy were ensured by limiting access to information exclusively to the researchers and assigning a random number to each participant. Participants had the autonomy to review the questionnaire before participating, and informed consent was obtained via signature.

## 3 Results

### 3.1 Population characteristics

**Figure 1:**
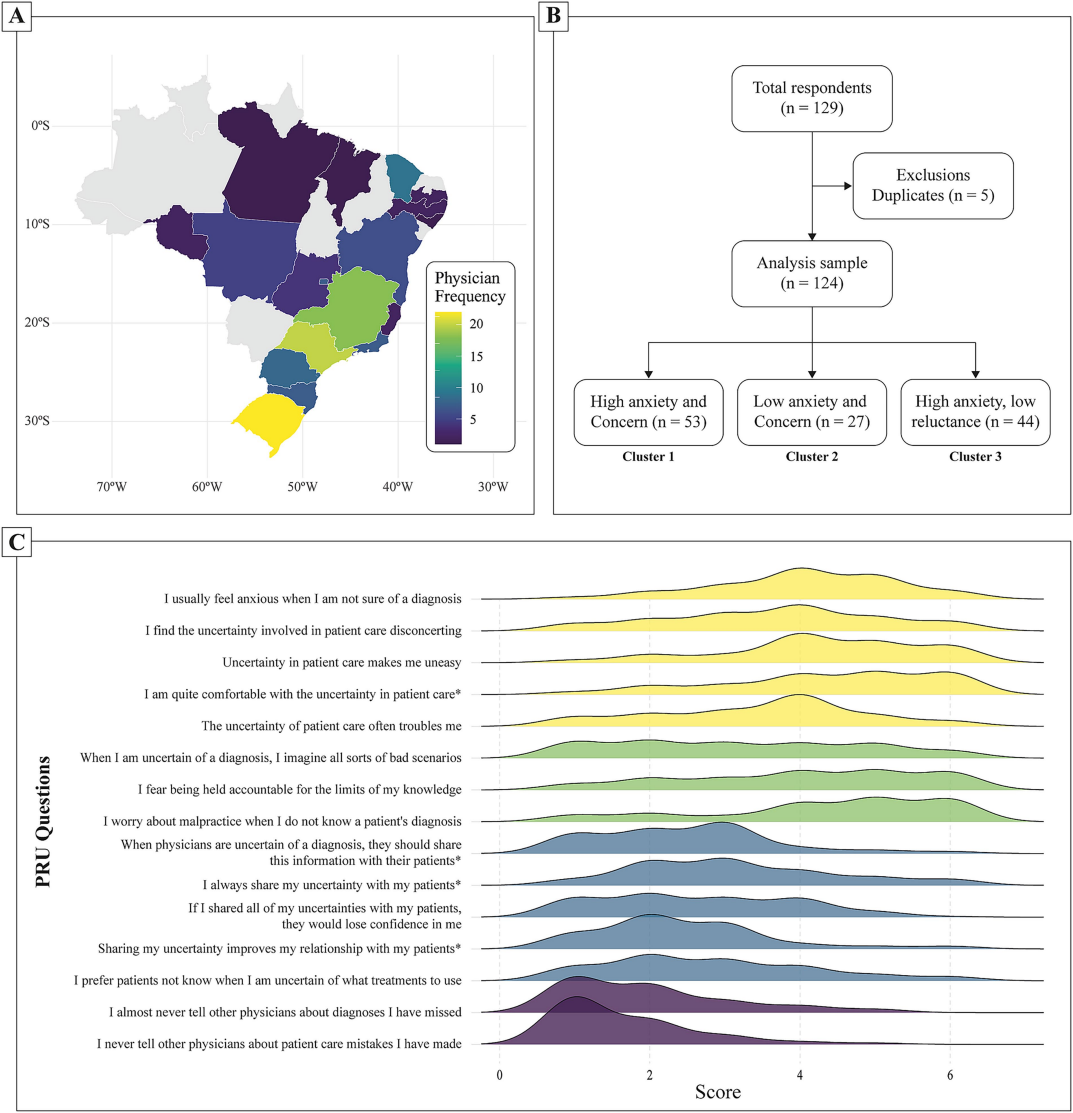
Panel of geographic distribution, cluster analysis, and response patterns. **(A)** geographic distribution of participating emergency medicine residents across Brazil. The color gradient indicates the frequency of physicians from each state, with darker shades representing lower participation. **(B)** Study’s flowchart. **(C)** Distribution of responses for each item in the Physicians’ Reactions to Uncertainty questionnaire. The *x*-axis represents the score on a Likert scale from 0 to 6, while the y-axis lists the individual questionnaire items. The density curves indicate the frequency of responses. The repetition of the colors represents the four domains evaluated by the questionnaire. Specific items (*) are reverse scored to account for inverse wording.

A total of 124 EMRs participated in this study (Figures 1A,B and Table 1). The median age was 28 years (IQR: 26–31 years), with a near-equal distribution between female (52%) and male (48%) respondents. The majority of the participants (49%) were in their first year of residency (R1), followed by 27% in their second year (R2) and 23% in their third year (R3). The majority of the residents had no prior medical residency (92%) or other healthcare-related degrees (94%) (Table 1). Geographically, respondents were distributed across all regions of Brazil, with the largest proportion from the Southeast (38%), followed by the South (30%), Northeast (17%), Midwest (13%), and North (2%) regions (Figure 1A). The median time from graduation to residency was 3 years (IQR: 1–3 years).

**Table 1:** Population characteristics.

Characteristic	*N* = 124
Age, years	28.0 [26.0, 31.0]
Sex	
Female	65 (52%)
Male	58 (47%)
No answer	1 (0.8%)
Time from graduation to residency	2.00 [1.00, 3.00]
Year of residence	
R1	61 (49%)
R2	34 (27%)
R3	29 (23%)
Previous graduation in healthcare	
No	117 (94%)
Yes	7 (5.6%)
Previous medical residency	
No	114 (92%)
Yes	10 (8.1%)
Region	
Midwest	16 (13%)
Northeast	21 (17%)
North	3 (2.4%)
Southeast	47 (38%)
South	37 (30%)

Description of the population characteristics. Median [Q1, Q3]; *n* (%). Abbreviations: R1, first year of residence; R2, second year of residence; R3, third year of residence.

The median total PRU score among respondents was 49.0 (IQR: 41.0–57.0). When analyzed by dimension, anxiety due to uncertainty (D1) had a median score of 21.0 (IQR: 17.8–23.0), concern about bad outcomes (D2) had a median of 12.0 (IQR: 8.75–14.0), reluctance to disclose uncertainty to patients (D3) had a median of 13.0 (IQR: 9.75–17.0), and unwillingness to disclose mistakes to other physicians (D4) had a median of 3.0 (IQR: 2.0–5.0). Responses varied widely, with certain statements showing a concentration of high agreement, such as concerns about losing patient trust when disclosing uncertainties and anxiety related to not knowing a diagnosis. Items reflecting a willingness to share uncertainties with colleagues and patients had more evenly distributed responses (Figure 1C).

**Figure 2:**
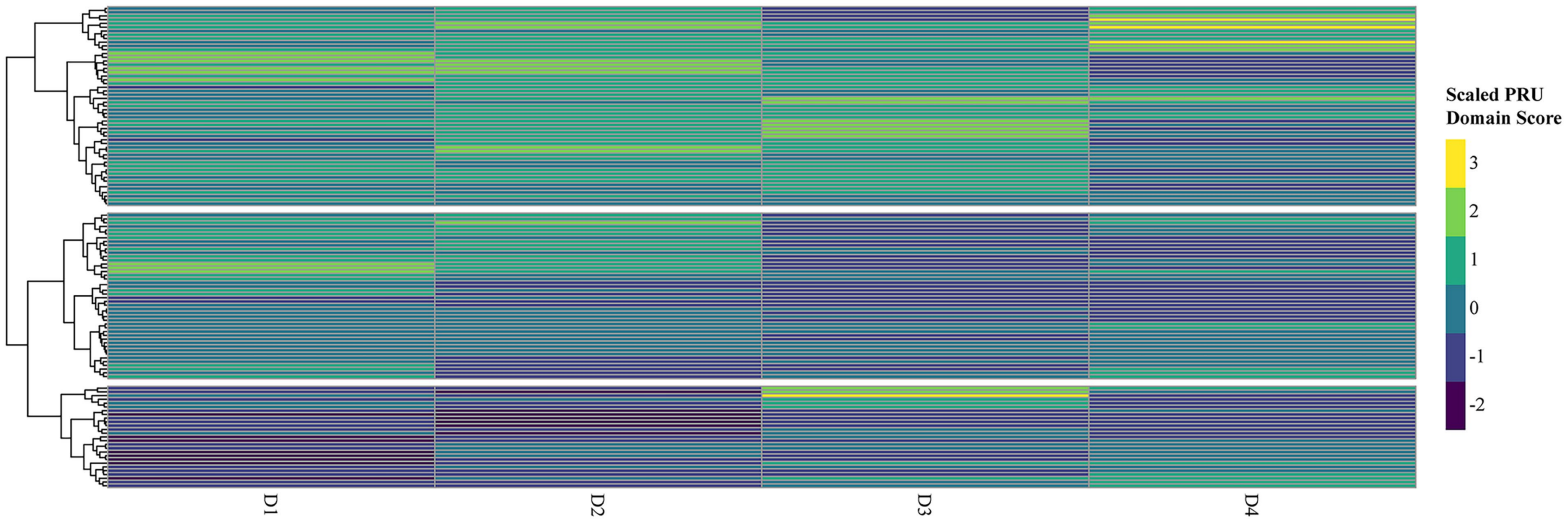
Scaled PRU domain scores with hierarchical clustering. Heatmap of standardized (Z-scored) domain means from the Physicians’ Reactions to Uncertainty scaled scores across the four dimensions: anxiety due to uncertainty (D1), concern about bad outcomes (D2), reluctance to disclose uncertainty to patients (D3), and reluctance to disclose mistakes to other physicians (D4). The color scale ranges from blue (lower concern) to yellow (greater concern). Rows represent individual respondents; columns represent PRU domains. Hierarchical clustering was performed using Euclidean distance and Ward. D2 linkage, revealing three distinct subgroups.

Hierarchical clustering analysis identified three distinct subgroups of residents based on their PRU scores (Figure 2). Cluster 1 (High PRU, N = 53): Residents in this group had significantly higher total PRU scores (median: 58.0, IQR: 52.0–61.0), with particularly elevated anxiety from uncertainty (D1: 22.0, IQR: 20.0–24.0) and concern about bad outcomes (D2: 14.0, IQR: 13.0–16.0). Cluster 2 (Low PRU, *N* = 27): This group exhibited the lowest overall PRU scores (median: 35.0, IQR: 30.0–41.5), with notably lower anxiety (D1: 13.0, IQR: 11.0–14.0) and concern about bad outcomes (D2: 8.0, IQR: 4.5–8.5). Cluster 3 (Moderate PRU, *N* = 44): Residents in this group had intermediate PRU scores (median: 46.0, IQR: 41.0–48.2), with similar anxiety (D1: 21.0, IQR: 19.0–23.0) but lower reluctance to disclose uncertainty (D3: 10.0, IQR: 9.0–12.0) compared to Cluster 1 (Table 2). For comparison, the original table format, presenting column percentages (i.e., percentage of each cluster by demographic group), is available in Supplementary Table S1.

**Table 2:** Distribution of participant characteristics across PRU clusters.

Characteristics	[ALL] *N* = 124	[Cluster 1] High anxiety and concern (*N* = 53)	[Cluster 2] Low anxiety and concern (*N* = 27)	[Cluster 3] High anxiety, low concern (*N* = 44)	*p*-value
Age, years	28.0 [26.0; 31.0]	28.0 [26.0;30.0]	29.0 [27.0;32.0]	27.0 [25.0;30.2]	0.047
Sex					0.380
Female	65 (52.4%)	32 (49.2%)	13 (20.0%)	20 (30.8%)	
Male	58 (46.8%)	21 (36.2%)	14 (24.1%)	23 (39.7%)	
No answer	1 (0.81%)	0 (0.00%)	0 (0.00%)	1 (100%)	
Country region					0.713
Midwest	16 (12.9%)	6 (37.5%)	2 (12.5%)	8 (50.0%)	
Northeast	21 (16.9%)	7 (33.3%)	7 (33.3%)	7 (33.3%)	
North	3 (2.42%)	2 (66.7%)	0 (0.00%)	1 (33.3%)	
Southeast	47 (37.9%)	21 (44.7%)	12 (25.5%)	14 (29.8%)	
South	37 (29.8%)	17 (45.9%)	6 (16.2%)	14 (37.8%)	
Time for graduation to residence, years	2.00 [1.00; 3.00]	1.00 [1.00; 2.00]	2.00 [1.00; 3.00]	1.00 [1.00; 2.00]	0.046
Current year of residence					0.054
R1	61 (49.2%)	29 (47.5%)	8 (13.1%)	24 (39.3%)	
R2	34 (27.4%)	15 (44.1%)	7 (20.6%)	12 (35.3%)	
R3	29 (23.4%)	9 (31.0%)	12 (41.4%)	8 (27.6%)	
Previous graduation in healthcare					0.422
No	117 (94.4%)	51 (43.6%)	24 (20.5%)	42 (35.9%)	
Yes	7 (5.65%)	2 (28.6%)	3 (42.9%)	2 (28.6%)	
Previous medical residency					0.055
No	114 (91.9%)	49 (43.0%)	22 (19.3%)	43 (37.7%)	
Yes	10 (8.06%)	4 (40.0%)	5 (50.0%)	1 (10.0%)	
PRU Scores					
TOTAL	49.0 [41.0; 57.0]	58.0 [52.0; 61.0]	35.0 [30.0; 41.5]	46.0 [41.0; 48.2]	<0.001
D1	21.0 [17.8; 23.0]	22.0 [20.0; 24.0]	13.0 [11.0; 14.0]	21.0 [19.0; 23.0]	<0.001
D2	12.0 [8.75; 14.0]	14.0 [13.0; 16.0]	8.00 [4.50; 8.50]	10.0 [8.75; 13.0]	<0.001
D3	13.0 [9.75; 17.0]	17.0 [15.0; 19.0]	12.0 [9.50; 16.0]	10.0 [9.00; 12.0]	<0.001
D4	3.00 [2.00; 5.00]	3.00 [2.00; 6.00]	3.00 [2.00; 4.00]	3.00 [2.00; 4.00]	0.149

Distribution of sociodemographic, academic, and PRU-related variables across the different clusters identified in the study. For categorical variables, the table presents the proportion of participants within each subgroup allocated to each cluster (row percentages). The *p*-values correspond to the Kruskal–Wallis test for comparing continuous variables and the chi-squared test for comparing the distribution of categorical variables. R1, first year of residence; R2, second year of residence; R3, third year of residence; PRU, Physicians’ Reactions to Uncertainty; D1, anxiety from uncertainty; D2, concern about bad outcomes; D3, reluctance to disclose uncertainty to patients; D4, reluctance to disclose mistakes to other physicians.

### 3.2 Determinants of PRU scores

Multivariable linear regression models were used to assess factors associated with PRU scores and their subdomains (Table 3). Male residents had significantly lower total PRU scores (*β* = −4.13, 95% CI: −7.83 to −0.42, *p* = 0.029), with the strongest difference observed in the anxiety from uncertainty domain (*β* = −1.94, 95% CI: −3.61 to −0.27, *p* = 0.023). Third-year residents had significantly lower total PRU scores (*β* = −7.62, 95% CI: −12.86 to −2.37, *p* = 0.005), with the most pronounced reduction in anxiety (*β* = −2.88, 95% CI: −5.24 to −0.52, *p* = 0.017) and concern about bad outcomes (*β* = −1.87, 95% CI: −3.87 to −0.13, *p* = 0.066). Previous healthcare-related education and prior residency experience did not show significant associations with total PRU scores or subdomains (*p* > 0.05). No factors were associated with reductions in domains other than anxiety.

**Table 3:** Characteristics of EMRs associated with PRU scores.

Predictors	D1			D2			D3			D3			TOTAL		
	Estimates	95% CI	*p*-value	Estimates	95% CI	*p*-value	Estimates	95% CI	*p-*value	Estimates	95% CI	*p-*value	Estimates	95% CI	*p*-value
(Intercept)	20.78	11.44 – 30.13	<0.001	15.81	7.91 –23.72	<0.001	10.63	0.43 –20.84	0.041	10.63	0.43 –20.84	0.041	51.56	30.80 –72.32	<0.001
Age, years	0.1	−0.29 – 0.48	0.619	−0.06	−0.39 – 0.26	0.704	0.19	−0.24 – 0.61	0.386	0.19	−0.24 – 0.61	0.386	0.21	−0.65 – 1.06	0.633
Sex [Male]	−1.94	−3.61 – −0.27	0.023	−1.11	−2.52 – 0.30	0.123	−1.29	−3.11 – 0.54	0.165	−1.29	−3.11 – 0.54	0.165	−4.13	−7.83 – −0.42	0.029
Time from graduation to residence, years	−0.48	−1.26 – 0.29	0.22	−0.48	−1.13 – 0.18	0.152	−0.41	−1.26 – 0.43	0.335	−0.41	−1.26 – 0.43	0.335	−1.5	−3.22 – 0.21	0.086
Year of residence															
Second year	−1.77	−3.81 – 0.27	0.088	−0.7	−2.43 – 1.03	0.423	−0.54	−2.77 – 1.69	0.631	−0.54	−2.77 – 1.69	0.631	−2.72	−7.26 – 1.81	0.236
Third year	−2.88	−5.24 – −0.52	0.017	−1.87	−3.87 – 0.13	0.066	−2.37	−4.95 – 0.21	0.071	−2.37	−4.95 – 0.21	0.071	−7.62	−12.86 – −2.37	0.005
Previous graduation in healthcare [Yes]	−2.82	−6.84 – 1.19	0.166	0.04	−3.36 – 3.43	0.982	−0.9	−5.29 – 3.48	0.684	−0.9	−5.29 – 3.48	0.684	−4.78	−13.70 – 4.14	0.291
Previous residence [Yes]	−0.59	−4.49 – 3.32	0.767	1.66	−1.64 – 4.96	0.321	3.37	−0.89 – 7.63	0.12	3.37	−0.89 – 7.63	0.12	5.65	−3.02 – 14.32	0.199
Observations	124			124			124			124			124		
R^2^/R^2^ adjusted	0.179/0.122			0.122/0.061			0.068/0.003			0.068/0.003			0.175/0.118		

Values represent *β* coefficients with 95% CIs, obtained from regression models. The *p*-values indicate the statistical significance of the associations. Negative *β* values suggest a lower PRU score in relation to the reference group, while positive values indicate a higher PRU score. EMRs, emergency medicine residents; PRU, Physicians’ Reactions to Uncertainty; D1, anxiety from uncertainty; D2, concern about bad outcomes; D3, reluctance to disclose uncertainty to patients; D4, reluctance to disclose mistakes to other physicians.

## 4 Discussion

This study demonstrated that experience, both in terms of years of residency and prior training, significantly impacts physicians’ reactions by reducing perceived uncertainty and mitigating its emotional and cognitive effects. This, in turn, can directly influence clinical decisions, behaviors, and overall well-being. These findings are consistent with previous studies that emphasize the role of formal education, clinical practice, and cumulative experiences in developing strategies for managing uncertainty in patient care decisions (22–24).

We found that while certain demographic and professional characteristics significantly influenced residents’ responses to uncertainty, others did not show such an association. Specifically, male residents and those in their third year of residency had significantly lower PRU scores, particularly in the anxiety domain, indicating less emotional distress when facing uncertainty. In contrast, factors such as prior healthcare-related education or previous residency experience were not significantly associated with PRU scores or any of its subdomains. Although previous studies have linked age and gender to tolerance of uncertainty (25, 26), there is no consensus in the literature regarding these influences (9, 15, 18, 27). Our findings suggest that gender differences are limited to the emotional response (anxiety), rather than overall levels of uncertainty. However, it is also important to consider how patients perceive uncertainty disclosure. Previous studies have shown that patients may respond more negatively to uncertainty expressed by female physicians, indicating a potential gender bias in how such communication is received. This dynamic may further influence physicians’ willingness to share uncertainty with patients, particularly among female professionals (28). Similar to a previous study on surgical residents (29), age was not associated with significant differences in the degree of uncertainty.

It is important to note that uncertainty is inherent in everyday medical practice, affecting everyone from medical students to experienced physicians. During residency, professionals face considerable levels of uncertainty in clinical decision-making. The findings that previous healthcare-related education and prior residency experience did not significantly influence PRU scores reinforce the idea that managing uncertainty is a skill honed through direct clinical exposure rather than through prior theoretical training. Repetition of practical tasks and exposure to a variety of complex clinical scenarios can enhance residents’ abilities to confidently and efficiently handle uncertain situations (9, 22, 29, 30). This contrasts with the significant reductions in PRU scores seen among third-year residents, suggesting that hands-on experience and exposure to a variety of clinical situations are more critical in developing the ability to handle uncertainty effectively.

Another significant finding is that managing uncertainty in decision-making appears more challenging for first-year residents, as they are in the early stages of their professional careers (1, 22, 25, 26, 31, 32). This vulnerability may be intensified by high levels of stress and unhealthy coping behaviors frequently reported among healthcare students, such as increased alcohol consumption, which has been linked to poor academic performance and may impair cognitive function and emotional regulation (33). Additionally, first-year residents often exhibit higher levels of skepticism about their own abilities (22). These factors may exacerbate difficulties in clinical reasoning and decision-making under uncertainty (33). This challenge is compounded by conditions that negatively affect cognitive performance and emotional regulation, further hindering residents’ ability to manage diagnostic uncertainty and potentially impacting their clinical reasoning and decision-making processes.

These observations reinforce that uncertainty is a universal and relevant factor in medical practice, affecting professionals at different stages of their careers in distinct ways. Therefore, it is crucial for residency programs to incorporate strategies that address uncertainty and promote professional growth over time. This is supported by prior findings showing that emergency medicine residents themselves recognize the need for more structured training on how to communicate diagnostic uncertainty effectively with patients (34). Qualitative evidence also suggests that clinicians facing diagnostic uncertainty in complex scenarios, such as first-trimester bleeding, rely heavily on prior experience and communication skills to manage emotional distress and guide patient expectations, highlighting the need for intentional development of these competencies during training (35). Additionally, initiatives specifically designed to prepare medical students to communicate uncertainty in emergency settings have shown promise, suggesting that these skills can and should be developed early in medical training (36).

Moreover, an inability to manage uncertainty can cause distress among residents and negatively impact patients, potentially leading to excessive diagnostic testing and higher hospital admission rates (9, 23). Additionally, intolerance to uncertainty is linked to negative consequences such as stress, anxiety, and burnout among healthcare professionals (31). These findings have driven efforts to deepen the understanding of how individuals experience and manage uncertainty in the complex domain of healthcare. Therefore, recognizing and addressing medical uncertainty and developing strategies to cope with it are essential clinical skills for medical students and residents (23, 30). Tolerance to uncertainty is a crucial professional competency in medicine that can be fostered through educational programs addressing effective communication strategies to discuss uncertainties, thus reducing the negative reactions under these conditions (24, 37–39).

In summary, exploring the understanding and approach to uncertainty in clinical practice is a crucial step in the medical training process, despite challenges in objectively quantifying it (9, 15, 23). The inferences presented in this study may have significant implications for the training of emergency medicine residents. Incorporating teaching strategies that help first-year residents develop stress management, anxiety reduction, and problem-solving skills in uncertain situations can benefit their professional development. Additionally, understanding the role of prior education in managing uncertainty can help identify more personalized approaches in medical training, contributing to a more comprehensive and effective education.

The findings reported here provide valuable information, especially considering that the data collection period coincided with the coronavirus disease 2019 (COVID-19) pandemic, a global health crisis that not only intensified existing challenges in medical practice but also introduced new and significant sources of uncertainty. These included uncertainties related to diagnosis (e.g., limited test availability), evolving treatment protocols, and prognosis (40). The pandemic amplified stress and workload among healthcare professionals, which may have influenced residents’ experiences with uncertainty (41). Future studies should consider this context when analyzing and interpreting these findings.

While our study included participants from all regions of Brazil, the absence of complete demographic data for all EM residents (e.g., gender distribution across programs) prevents us from fully assessing the representativeness of our sample. According to the Medical Demography in Brazil 2025 (42), EM is a relatively new specialty, with 917 physicians currently in training (R1–R3) and one of the youngest profiles among all medical fields (36% aged ≤35 years; average age 39.6 ± 9 years; 62.8% male). In our sample, the regional distribution (38% Southeast, 30% South, 17% Northeast, 13% Midwest, and 2% North) mirrors the national distribution of EM programs. Still, the snowball sampling method may have introduced selection bias, as recruited participants may share similar characteristics or belong to the same social groups.

Additionally, although this study provides a quantitative overview of uncertainty among EMRs, the complexity of this phenomenon may not be fully captured by quantitative tools alone. Qualitative methods, such as in-depth interviews or focus groups, could offer richer insights into the underlying reasons for residents’ responses and perceptions, as well as the contextual factors influencing their decision-making under uncertainty.

Finally, the cross-sectional nature of the study prevents the determination of causal relationships between the studied variables, allowing only associations to be identified. Nonetheless, these findings offer valuable insights that can help mitigate the negative impacts of uncertainty on decision-making in patient care.

## 5 Conclusion

EMRs experience significant uncertainty in decision-making, with the highest levels observed in anxiety and reluctance to disclose uncertainty to patients. First-year residents, as well as those without prior healthcare education or residency experience, are particularly affected. Male and more experienced residents demonstrated lower levels of uncertainty, especially in the anxiety domain. This study highlights the influence of experience in managing uncertainty among emergency medicine residents, showing that residency duration and prior training directly impact their reactions, clinical decisions, and overall well-being. In addition to contributing to a deeper understanding of uncertainty in medical practice, this research underscores the need to develop effective strategies to address it, aiming not only for patient safety but also for the well-being of healthcare professionals.

## Data Availability

The raw data supporting the conclusions of this article will be made available by the authors upon reasonable request, without undue reservation.
