# Supplementary material for: Navigating uncertainty in patient care: a closer look at emergency medicine residents in Brazil
Source: Front Med (Lausanne). 2025 Sep 10;12:1578575. doi: 10.3389/fmed.2025.1578575 (PMC12457425; doi:10.3389/fmed.2025.1578575)
Supplement: Supplementary file 1 [file Table_1.DOCX]

Supplementary table 1. Characteristics of the Study Participants, Stratified by Cluster

| **Characteristics** | **[ALL] N=124** | **[Cluster 1]**  **High Anxiety and Concern**  **(N=53)** | **[Cluster 2]**  **Low Anxiety and Concern**  **(N=27)** | **[Cluster 3]**  **High Anxiety, low Concern**  **(N=44)** | **p-value** |
| --- | --- | --- | --- | --- | --- |
| Age, years | 28.0 [26.0;31.0] | 28.0 [26.0;30.0] | 29.0 [27.0;32.0] | 27.0 [25.0;30.2] | 0.047 |
| Sex |  |  |  |  | 0.380 |
| Female | 65 (52.4%) | 32 (60.4%) | 13 (48.1%) | 20 (45.5%) |  |
| Male | 58 (46.8%) | 21 (39.6%) | 14 (51.9%) | 23 (52.3%) |  |
| No answer | 1 (0.81%) | 0 (0.00%) | 0 (0.00%) | 1 (2.27%) |  |
| Country region |  |  |  |  | 0.713 |
| Midwest | 16 (12.9%) | 6 (11.3%) | 2 (7.41%) | 8 (18.2%) |  |
| Northeast | 21 (16.9%) | 7 (13.2%) | 7 (25.9%) | 7 (15.9%) |  |
| North | 3 (2.42%) | 2 (3.77%) | 0 (0.00%) | 1 (2.27%) |  |
| Southeast | 47 (37.9%) | 21 (39.6%) | 12 (44.4%) | 14 (31.8%) |  |
| South | 37 (29.8%) | 17 (32.1%) | 6 (22.2%) | 14 (31.8%) |  |
| Time for graduation to residence, years | 2.00 [1.00;3.00] | 1.00 [1.00;2.00] | 2.00 [1.00;3.00] | 1.00 [1.00;2.00] | 0.046 |
| Current year of residence |  |  |  |  | 0.054 |
| R1 | 61 (49.2%) | 29 (54.7%) | 8 (29.6%) | 24 (54.5%) |  |
| R2 | 34 (27.4%) | 15 (28.3%) | 7 (25.9%) | 12 (27.3%) |  |
| R3 | 29 (23.4%) | 9 (17.0%) | 12 (44.4%) | 8 (18.2%) |  |
| Previous graduation in healthcare |  |  |  |  | 0.422 |
| No | 117 (94.4%) | 51 (96.2%) | 24 (88.9%) | 42 (95.5%) |  |
| Yes | 7 (5.65%) | 2 (3.77%) | 3 (11.1%) | 2 (4.55%) |  |
| Previous medical residency |  |  |  |  | 0.055 |
| No | 114 (91.9%) | 49 (92.5%) | 22 (81.5%) | 43 (97.7%) |  |
| Yes | 10 (8.06%) | 4 (7.55%) | 5 (18.5%) | 1 (2.27%) |  |
| PRU Scores |  |  |  |  |  |
| TOTAL | 49.0 [41.0;57.0] | 58.0 [52.0;61.0] | 35.0 [30.0;41.5] | 46.0 [41.0;48.2] | <0.001 |
| D1 | 21.0 [17.8;23.0] | 22.0 [20.0;24.0] | 13.0 [11.0;14.0] | 21.0 [19.0;23.0] | <0.001 |
| D2 | 12.0 [8.75;14.0] | 14.0 [13.0;16.0] | 8.00 [4.50;8.50] | 10.0 [8.75;13.0] | <0.001 |
| D3 | 13.0 [9.75;17.0] | 17.0 [15.0;19.0] | 12.0 [9.50;16.0] | 10.0 [9.00;12.0] | <0.001 |
| D4 | 3.00 [2.00;5.00] | 3.00 [2.00;6.00] | 3.00 [2.00;4.00] | 3.00 [2.00;4.00] | 0.149 |

Table legend: Comparison between participants characteristics among the different clusters identified in the study. The p-values correspond to Kruskal-Wallis test, for the comparison of continuous variables, and chi-squared test, comparing the distribution of categorical variables. Abbreviations - R1: 1st year of residence; R2: 2nd year of residence; R3: 3rd year of residence; PRU: Physicians’ Reactions to Uncertainty; D1: Anxiety from uncertainty; D2: Concern about bad outcomes; D3: Reluctance to disclose uncertainty to patients; D4: Reluctance to disclose mistakes to other physicians.
